# Levels and trends of contaminants in humans of the Arctic

**DOI:** 10.3402/ijch.v75.33804

**Published:** 2016-12-13

**Authors:** Jennifer Gibson, Bryan Adlard, Kristin Olafsdottir, Torkjel Manning Sandanger, Jon Øyvind Odland

**Affiliations:** 1 Health Canada, Ottawa, ON, Canada; 2 Department of Pharmacology and Toxicology, University of Iceland, Reykjavik, Iceland; 3 Department of Community Medicine, UiT The Arctic University of Norway, Tromsø, Norway

**Keywords:** Arctic, contaminants, biomonitoring, POPs, metals, pregnant women

## Abstract

The Arctic Monitoring and Assessment Programme (AMAP) is one of the six working groups established under the Arctic Council. AMAP is tasked with monitoring the levels of contaminants present in the Arctic environment and people as well as assessing their effects on a continuous basis, and reporting these results regularly. Most of the presented data have been collected over the last 20 years and are from all eight Arctic countries. Levels of contaminants appear to be declining in some of the monitored Arctic populations, but it is not consistent across the Arctic. Most Arctic populations continue to experience elevated levels of these contaminants compared to other populations monitored globally. There are certain contaminants, such as perfluorinated compounds and polybrominated diphenyl ethers, which are still increasing in Arctic populations. These contaminants require more investigation to find out the predominant and important sources of exposure, and whether they are being transported to the Arctic through long-range transport in the environment.

Exposure to environmental contaminants through the traditional diet remains one of the risks to human health in the Arctic. Due to unique geographic and climatic characteristics, the Arctic has become a repository for contaminants transported long distances through the atmosphere and via ocean currents. Often persistent, these chemicals then bioaccumulate and biomagnify through Arctic food chains into the species that make up traditional food sources for many Arctic peoples. The traditional diet of these Arctic populations tends to rely on foraged plant matter, fish, and terrestrial and marine mammals for sustenance, as market foods are difficult to access or are not as nutrient-rich as traditional foods (1). Many of the marine mammals, some of which are top predators in the Arctic marine food web, and some fish species can be the most highly contaminated with persistent, bioaccumulative chemicals. Biomonitoring studies investigating the changing levels of contaminants in human populations are an essential part of the management of these risks, including the ability to analyse the risks and benefits for human populations which consume traditional food.

For several Arctic countries, there are now almost 20 years of biomonitoring data available to assess changes in contaminant concentrations. Persistent organic pollutants (POPs) and metals are still undergoing long-range transport to the Arctic and are bioaccumulating within the Arctic food chains relied upon for a socially, economically, culturally and nutritionally beneficial traditional food supply. However, declines are beginning to be detected in certain Arctic populations, and public health interventions have been instituted based on some of the biomonitoring results presented. Different contaminants are also being detected in the Arctic, indicating that new international risk management may be necessary.

## Methods

Since the first Arctic Monitoring and Assessment Programme (AMAP) Human Health expert meeting in 1991, considerable efforts have been made to ensure the measurement of high-quality human biomonitoring data for POPs and metals of concern to AMAP. The AMAP Human Health Assessment Group recommended in 2000 that a quality assessment (QA) programme needs to be established for POPs in human biological fluids (the AMAP Ring Test). The 2009 AMAP Human Health Assessment (2) described in detail the implementation and evolution of this external quality assessment scheme (EQAS), which has provided a means of comparing the quality of data produced by laboratories involved in the measurement of POPs in samples of human origin from Arctic countries (3).

Since its inception in 2001, the scope of the AMAP Ring Test has changed through addition or removal of different POPs. The scheme now considers 37 analytes [11 pesticides, 8 polybrominated diphenyl ethers (PBDEs), 9 polychlorinate biphenyl (PCBs), 6 perfluorinated compounds (PFCs), total lipids, cholesterol and triglycerides]. Several international EQAS, as well as a number of national schemes (2), already exist for metals in biological fluids.

It is important for AMAP to present spatial and temporal trends of exposure with confidence and to demonstrate that the trends are not influenced unduly by analytical uncertainty. The AMAP Ring Test has thus established criteria for good (within 20% of target) and acceptable (within 40% of target) EQAS performance. Although fluctuations in individual performance are expected, the proportion of laboratories showing excellent or good performance generally increased between 2001 and 2007, often with pronounced improvements for some analytes (2). Ongoing participation in the AMAP Ring Test (or another suitable intercomparison programme) is therefore highly encouraged, as this seems to have provided impetus for participating laboratories to examine and refine analytical procedures and organizational protocols necessary for meeting performance targets. All laboratories submitting data to this assessment were active participants in a QA/quality control (QC) programme (4).

The data for POPs, organochlorine (OCs) and PBDEs are expressed on a lipid weight basis (µg/kg plasma lipid) because these analytes accumulate in lipids, and lipid levels in plasma increase throughout pregnancy and vary in response to food intake. The data for POPs are expressed on a wet weight basis (µg/L plasma or serum) as they do not accumulate in lipids. The data for metals are expressed on a whole blood basis (µg/L whole blood). For full data sets and further details, see AMAP (4).

## Alaska

The Alaskan Native Maternal Organics Monitoring (AN MOM) programme began in 1998. Prospective repeat sampling of the same population resulted in recruitment of a cohort of 160 participants for the most recent sampling period (2009–2012), from the Yukon and Kuskokwim River Delta in southwestern Alaska.

Concentrations of POPs were lower in maternal samples from the 2009 to 2012 sampling period than that of previous periods (Table I). The pattern for *trans*-nonachlor, hexachlorobenzene (HCB) and PCB153 is less clear, as concentrations in the sample population from 2004 to 2006 were higher than those found in samples from 1999 to 2003. For *trans-*nonachlor and HCB, concentrations in 2009–2012 were lower than those of 2004–2006, but not yet as low as 1999–2003. For PCB153, the current concentrations are lower than those seen in the past two sampling periods, despite the higher concentrations in 2004–2006. The PBDEs were lower compared to the sampling in 2004–2006.

**Table I:** Concentrations of POPs and metals in Yup'ik maternal blood from Alaska

	1999–2003	2004–2006	2009–2012

Mean age sample size	26 (n=106)	26.4 (n=206)	26.5 (n=156)
* trans*-Nonachlor	5.3	21 (1.8–310)	9.9 (<LOD–107)
* *HCB	14	22 (2.4–188)	15.9 (2.7–98.8)
* *PCB153	17	24 (0.8–416)	14.8 (1.5–148)
* *PBDE47	na	26 (1.2–455)	19.8 (2.4–395)
* *PBDE99	na	6.3 (0.2–1,359)	4.5 (<LOD–186)

Mean age sample size	25 (n=43)	25 (n=75)	26.5 (n=160)

* *Total Hg	1.1	5	2.2
* *Pb	11	18	7.4
* *Cd	0.6	0.4	0.2
* *Se	na	na	181

Data presented as geometric means (range), calculated for the specified period of sampling, in µg/kg plasma lipid (PCBs and PBDEs), µg/L plasma (PFCs) or µg/L whole blood (metals). PBDE, polybrominated diphenyl ethers.

Comparison of the recent metals analysis with data reported by AMAP (2) shows that levels of cadmium (Cd), lead (Pb) and mercury (Hg) have decreased in Yup'ik mothers (Table I). Although the statistical significance of these trends was not measured, there has been a steady decline in Cd concentrations across the three sampling periods, and Pb and Hg concentrations in 2009–2012 are less than half the values of 2004–2006. Selenium (Se) is also reported for the first time. Levels reported in Yup'ik mothers are similar to those found in US females (all ages), which had a geometric mean of 188 µg/L blood Se in the National Health and Nutrition Examination Survey (NHANES) (5).

## Canada

Data presented include concentrations of contaminants among pregnant women in Nunavik through continued biomonitoring in the regions of Hudson Bay and the Ungava Coast. The 2007–2008 Inuit Health Survey provides coverage of three Inuit regions in northern Canada: the Inuvialuit Settlement Region, Nunavut and Nunatsiavut (6–8). Nunatsiavut is not an Arctic region, as it covers the northern coast of Labrador in the province of Newfoundland and Labrador, but it is an Inuit region and so is included in the study. Combined with the Nunavik Health Survey (2004), these studies provide biomonitoring data sets of contaminant levels among the Inuit across the Canadian North. The similar random sample study methodology used for these surveys, as outlined by Dewailly et al. (9), Saudny et al. (10) and Laird et al. (11), allows for a reliable comparison of biomonitoring data in northern Canada.

Concentrations of POPs among men in the Inuvialuit Settlement region and Nunavut and Nunatsiavut regions were usually higher than in all women, often by as much as two- or threefold (Table II). However, differences in POPs concentrations between men and all women studied in Nunavik did not show a clear trend by sex, and concentrations were often quite similar. Concentrations of Hg and Pb among men (Table II) were generally higher than among all women across the four Inuit regions of northern Canada, sometimes by as much as twofold. However, in Nunavik, women's Hg concentrations slightly exceeded that of men, although men reported consuming significantly higher amounts of traditional foods (by weight) (12). Selenium concentrations appeared similar between men and all women in each region.

**Table II:** Blood concentrations of POPs and metals in men and women across the Canadian Arctic

	Inuvialuit Settlement Region		Nunavut		Nunavik		Nunatsiavut	
				
	Men	Women	Men	Women	Men	Women	Men	Women
			
	2007–2008		2007–2008		2004		2007–2008	
Mean age (range)	46.2 (18–81)	42.6 (18–90)	42.2 (18–89)	40.7 (18–90)	37.0 (18–74)	37.3 (18–74)	45.7 (18–89)	43.4 (20–79)
Sample size (n)	92	187	650	977	408	506	98	165
*p,p’*-DDE	466 (9.4–8,189)	238 (13–3,251)	414 (1.4–4,337)	282 (1.5–7,727)	460 (13–7,000)	470 (27–8,300)	190 (18.9–2,834)	150 (13.1–2,789)
PCB153	148 (1.9–2,730)	62 (0.8–4,408)	195 (0.7–5,747)	106 (0.8–6,181)	190 (6.3–3,400)	160 (5.7–5,800)	112 (1.3–1,622)	61 (2.2–1,291)
Total Hg	5.6 (0.3–50)	4.1 (0.1–55)	9.4 (0.1–110)	7.9 (0.1–130)	9.2 (0.1–240)	12 (0.2–160)	4.2 (0.2–50)	2.8 (0.1–25)
Pb	44.5 (8.1–220)	27.6 (4.5–190)	46.1 (7.4–380)	32.2 (5.1–400)	46 (9.1–500)	34 (5.8–310)	40.1 (8.4–170)	22.4 (5.6–160)
Se	317 (160–1,300)	293 (150–1,200)	348 (130–2,800)	342 (85–2,500)	280 (130–3,500)	300 (120–2,400)	233 (150–1,500)	204 (140–960)

Data presented as geometric means (range), in lipid weight (µg/kg plasma lipid) for POPs and (µg/L whole blood) for metals.

Table III compares data for pregnant women in 2004, 2012 and 2013 for a select number of contaminants for which there are limited data over the past 20 years. Comparisons were made on a wet weight basis. Perfluorooctanesulfonic acid (PFOS) showed a distinct decline between 2004 and 2012. Selected metals data are presented here for pregnant Inuit women from Nunavik. Since 1992, significant declines in concentrations of metals have been observed among the Inuit in Nunavik (4). Mercury decreased by 59%, with an average annual decrease of 4%. The decrease in maternal blood Pb concentrations is less consistent than for Hg, showing an irregular pattern of increases and decreases. Selenium concentrations have decreased over time but not to the same degree as for Hg and Pb, although most recent Se concentrations appear to have risen slightly. Despite a significant decrease in consumption of traditional foods between 1992 and 2004 (13), concentrations of Se remain high in Nunavik.

**Table III:** Time series of POPs and metals concentrations in pregnant Inuit women from Nunavik, Canada

	2004	2012	2013
Mean age (range)	27 (18–42)	24 (18–39)	24 (18–41)
Sample size (n)	31	112	95
PFOS	9.8 (3.1–20)	3.9 (0.7–23)	na
PFOA	na	0.7 (0.2–2.4)	na
Total Hg	7.6 (1.2–30)	5.0 (0.2–40)	5.2 (0.3–32)
Pb	19 (5.8–85)	13 (2.7–230)	14 (4.2–62)
Se	270 (130–700)	320 (120–3,000)	300 (130–1,400)

Data presented as geometric means (range), in µg/L whole blood.

## Greenland

Greenland is home to an extensive biomonitoring network, and several studies have been carried out with the co-operation of the Greenlandic Inuit and other Greenlanders to monitor levels of contaminants in a population historically subject to some of the highest concentrations of POPs and metals in the Arctic.

The Inuit Health in Transition study found PCB153 in high concentrations in both men and women across all age groups and also noted that it increased significantly with increasing age. PCB153 was also present in high levels in the highest age groups (Table IV). This supports the well-known age-related bioaccumulation of organochlorine compounds. Among the OCs, HCB, *trans*-nonachlor and *p,p’*-Dichlorodiphenyldichloroethylene (DDE) appeared with the highest geometric mean levels in both sexes and all age groups (14, 15). The geometric mean of Hg ranged from 7.8 to 28.3 µg/L whole blood among men, and 8.3 to 22.1 µg/L whole blood among women (Table IV) (14, 15).

**Table IV:** Concentrations of PCBs and OCs (µg/kg plasma lipid) and metals (µg/L whole blood) among Inuit men and women in Greenland by age groups (years)

	24 (18–29)		41 (30–49)		61(<50)	
			
Mean age (range)	Men	Women	Men	Women	Men	Women
*trans*-Nonachlor	141 (7.6–820)	108 (11–940)	373 (9.8–5,000)	239 (5.0–1,500)	797 (12–3,500)	695 (31–4,000)
*p,p’*-DDE	559 (36–3,000)	444 (69–3,100)	1067 (97–16,000)	832 (10–6,600)	1816 (70–10,000)	1847 (130–27,000)
HCB	76.5 (10–360)	69.5 (13–490)	174 (8.0–2,300)	145 (6.0–910)	368 (13–1,800)	391 (36–1,700)
PCB153	253 (13–1,800)	170 (28–1,400)	545 (50–7,700)	382 (7.5–2,600)	1048 (54–4,600)	997 (93–6,700)
Total Hg	7.8 (0.1–160)	8.3 (0.1–120)	17 (0.4–490)	12.9 (0.1–230)	28.3 (0.1–400)	22.1 (0.1–320)
Se	188 (70–2,600)	203 (84–1,600)	280 (77–4,800)	266 (68–4,800)	352 (82–5,600)	374 (71–4,400)

Data presented as geometric means (range). Data from the Inuit Health in Transition study, 2005–2010 (14,15).

In the ACCEPT study, among 14 tested PCB congeners, three were in the higher concentration ranges, including PCB153 (Table VI). There were significant differences for PCB153 among regions. Pregnant women living in the East (Tasiilaq, n=3) and North (n=15) had higher PCB levels than that of women in other regions. Similar to PCBs, higher levels of OCs such as *trans*-nonachlor and HCB were found in pregnant Inuit women living in the East and North (16).

Among the PFCs measured, only six (PFOS, perfluorohexane sulfonate (PFHxS), perfluorooctanoic acid (PFOA), perfluorononanoic acid (PFNA), perfluorodecanoic acid (PFDA), perfluoroundecanoic acid (PFUnDA)) were detected in all samples. Although there is an age difference (ACCEPT mean age 27), these levels are lower (PFOS and PFOA, 10.5 and 1.2 µg/L, respectively) than the levels found in samples taken across the country during 1997–2006, where female (mean age 49) PFOS and PFOA geometric mean levels were 21 and 1.5 µg/L serum, respectively (17). Serum levels of PFOS in pregnant women from the North and East (Tasiilaq) were significantly higher than that of women living in the other regions. Women in the South and West had the lowest levels of PFCs (16).

Pregnant women from northern Greenland had significantly higher Hg levels compared to women from the other regions (Table V). However, no significant differences among regions were found for Pb and Cd. The trace element Se was detected in 100% of subjects. No significant differences among regions were found for Se (16).

**Table V:** Concentrations of POPs and metals in pregnant women from different regions in Greenland for 2010–2011 and 2013

	North	Disko Bay	West	South	East
Mean age (range)	28 (21–35)	27 (19–40)	27 (17–44)	28 (21–41)	33 (31–37)
No. individuals measured	n=15	n=45	n=118	n=13	n=3
*trans*-Nonachlor	85.1 (7.6–320)	57.7 (12–220)	35.2 (4.9–400)	41.2 (15–110)	184 (130–320)
*p,p’*-DDE	221 (18–990)	148 (35–430)	112 (16–1,100)	135 (39–430)	587 (300–1,300)
HCB	40.1 (5.8–130)	31.9 (13–100)	21.9 (6.4–170)	24.4 (10–41)	62.8 (47–110)
PCB153	99.2 (8.9–950)	62.4 (17–210)	53.7 (11–400)	63.1 (23–180)	288 (160–680)
PFOS	15.8 (4.7–50.7)	12 (3.7–32.8)	9.8 (2.5–61.3)	7.8 (3.2–18)	15.8 (6.1–26.5)
	n=14	n=49	n=121	n=15	n=3
Total Hg	8.0 (2.0–50)	4.0 (1.0–10)	3.1 (0–70)	4.0 (0.2–16)	7.0 (3.0–16)
Pb	7.0 (1.0–40)	7.0 (2.0–30)	7.0 (1.0–50)	7.0 (3.0–30)	6.0 (1.0–20)
Cd	0.5 (0–4.0)	1.0 (0–10)	0.9 (0–7.4)	1.1 (0–4.5)	0.7 (0–0.7)
Se	160 (60–1,040)	110 (60–360)	120 (40–2,660)	110 (70–250)	140 (90–190)

Data presented as geometric means (range). Data from the ACCEPT project. PCBs, OCs and PBDEs in µg/kg plasma lipid. PFCs in µg/L serum. Metals in µg/L whole blood (16).

## Iceland

In these studies, when visiting a medical centre, 50 pregnant women were invited to participate in biomonitoring sampling. This activity took place every 5 years in Iceland (2, 18, 19). A similar suite of contaminants were measured in each study, and all samples were collected from pregnant women during their third trimester (Olafsdottir, personal communication, 2014). Concentrations of metals in these maternal blood samples are not available. Reykjavik data are compared with data aggregated from all Iceland sampling done in previous years, as it was determined that the city of the mother's residence did not appear to influence measured contaminant levels. Iceland is considered to have a socially and culturally homogenous population (2).

The contaminants sampled in the 2009 cohort included brominated flame retardants and PFCs in addition to POPs. These data were not available in previous years. While the higher brominated congeners were detected in some participants (PBDE99, PBDE100 and PBDE153), the geometric mean of the population was below the detection limit (<1.3 µg/kg plasma lipid). However, the lowest brominated congener, PBDE47, was found up to a maximum of 21 µg/kg plasma lipid, and the geometric mean was higher than the detection limit, at 1.7 µg/kg plasma lipid. The PFCs, PFOS and PFOA were also analysed for the first time in this population, and levels were above the detection limit. There was an overall decrease in levels of contaminants between 1995 and 2009. *p,p’*-DDE and PCB153 have strong decreasing trends from 1995 to 2009. *Trans*-nonachlor shows a slight increase between 1995 and 1999 before a decrease occurs (Table VI).

**Table VI:** Trends in blood concentrations of POPs (µg/kg plasma lipid) in pregnant Icelandic women in their third trimester

	Reykjavik	All Iceland	All Iceland	Reykjavik
	1995	1999	2004	2009
Mean age (range)	30 (18–41)	28.7 (20–42)	30.3 (20–40)	30.4 (21–43)
Sample size, mean parity	n=40; p=1.9	n=39; p=1.9	n=40; p=1.8	n=33; p=1.7
*trans*-Nonachlor	12 (3.8–50)	15 (6.4–47)	7.1 (1.3–29)	6.7 (3.6–15.5)
*p,p’*-DDE	113 (42–514)	100 (33–306)	54 (19–226)	36 (12.1–139)
PCB153	68 (26–158)	60 (24–143)	40 (19–98)	34 (18–108)
PBDE47	–	–	–	1.7 (<1.3–21)
PBDE99	–	–	–	<1.3 (<1.3–3.7)
PBDE100	–	–	–	<1.3 (<1.3–5.0)
PBDE153	–	–	–	<1.3 (<1.3–3.9)
PFOS	–	–	–	6.2 (4.2–13)^a^
PFOA	–	–	–	4.8 (1.4–40)^a^

Data presented as geometric means (range). Lipid normalization of data in 1999 and 2004 based on average lipid concentrations from 1995. PFOS and PFOA in µg/L plasma.

a n=10. For statistical purposes, values <1.3 (the limit of detection, LOD) were replaced by LOD/2. PBDE, polybrominated diphenyl ethers.

## Faroe Islands

Researchers in the Faroe Islands have undertaken several biomonitoring cohort studies over the past 25 years, in response to the growing awareness of environmental contaminants and their possible effects on human health. The data presented in this section represent several follow-ups of Cohorts 1, 2, 3 and 5 (20–23); Weihe, personal communication, 2014).

There is an increase in levels of *p,p’*-DDE from cord blood to the sampling event at 13 years of age (Table VII), and a decrease from 13 years of age to the most recent sampling event, when cohort participants were 22 years of age. Changes in PCB levels show a decrease from cord blood to the sampling event at 22 years of age. PFCs were only sampled at the 7-year follow-up; therefore, recent changes are not known. Comparing 7-year-old children from Cohort 1 with 7.5-year-old children from Cohort 3 (Table VIII) shows the difference that approximately 20 years of dietary advice can make in addressing elevated levels of Hg, with measured levels decreasing from 8.36 to 1.99 µg/L whole blood. Total Hg levels decreased over time in the Cohort 1 sampling population.

**Table VII:** Time series of blood POPs concentrations from the Faroe Islands Cohort 1 (1986–1987)

	1986–1987	1993–1994	2000–2001	2008–2009
Mean age (range)	Cord blood	6.9 (6.3–8.3)	13.8 (12.8–15.2)	22.1 (20.9–23.7)
Sample size	n=1,022	n=922	n=792	n=849
*p,p’*-DDE	270 (4.2–4,487)	–	468 (25.4–8,050)	122 (5.4–3,257)
PCB153	130 (0.3–1,127)	–	–	93 (8.2–1,006)
PFOS	–	31.1 (7.2–96.9)	–	–
PFOA	–	5.4 (1.3–17.3)	–	–
Total Hg	22.3 (0.9–350)	8.4 (0.1–62.8)	4.1 (0.3–39.8)	2.5 (0.1–46.3)
Pb	15.8 (1.0–110)	–	–	–

All participants are Faroese children born between 1986 and 1987. Data presented as geometric means (range). POPs in µg/kg plasma lipid. PFCs and metals in µg/L whole blood.

**Table VIII:** Time series of blood POPs concentrations from the Faroe Islands Cohort 3 (1998–2000)

	1998–2000	2000–2001	2002–2005	2005–2007	2011–2012
	Mothers	Children	Children	Children	Children
Mean age (range)	28 (16–44)	1.5 (1.3–1.8)	5.0 (4.8–5.2)	7.5 (7.0–7.9)	13.2 (12.6–14.3)
Sample size	n=475	n=115	n=555	n=498	n=526
*p,p’*-DDE	538 (43.1–11,414)	613 (68–10,265)	476 (38.1–6,631)	270 (20–4,190)	92.8 (1.5–2,738)
PCB153	274 (36.7–320)	–	257 (17.6–2,162)	152 (6.7–3,223)	86 (1.6–924)
PFOS	27.4 (9.4–68.8)^a^	–	16.7 (3.3–48.2)	15.3 (5.6–35.5)	6.6 (1.0–16.6)
PFOA	3.2 (0.8–8.4)^a^	–	4.1 (0.8–15.4)	4.5 (1.7–19.2)	2.0 (0.6–6.1)
Total Hg	12.4 (1.6–193)^b^	–	2.6 (0.0001–36.5)	2.0 (0.1–58)	–
Pb	–	–	–	6.2 (0.02–47.7)	–

All participants were Faroese women with their children born between 1998 and 2000. Data presented as geometric means (range). POPs in µg/kg plasma lipid. PFCs and metals in µg/L whole blood.

a Mean age (range)=30.2 (16.7–43.2), n=618;

b Cord blood.

Cohort 2 has two data points available: that of the maternal blood at parturition in 1994–1995 and that of the children's blood at an average age of 7.5 years (Table IX). *p,p’*-DDE levels were not determined for the children. Almost all POPs levels found in the children at 7.5 years of age are lower than those for maternal blood at the time of giving birth. Total Hg and Pb levels did not substantially change between 1986–1987 and 1994–1995.

**Table IX:** Time series of blood POPs concentrations from the Faroe Islands Cohort 2 (1994–1995)

	1994–1995	2001–2002
	Mothers	Children
Mean age (range)	28 (16–44)	7.5 (7.4–7.8)
Sample size	n=182	n=158
*p,p’*-DDE	725 (201–8,038)	–
PCB153	265 (10–3,933)	176 (11.6–1,032)
Total Hg	21 (1.9–102)^a^	3.2 (0.1–22.1)
Pb	10.4 (1.2–41.4)^a^	–

All participants were Faroese women with their children born between 1994 and 1995. Data presented as geometric means (range). POPs in µg/kg plasma lipid. Metals in µg/L whole blood.

a Cord blood.

The intent of Cohort 3 was to follow up on the possible effects of PCBs and other lipophilic contaminants, as dietary advice had decreased the potential for MeHg exposure (Table VIII) (24). It can be seen that the child's levels of *p,p’*-DDE slightly exceeded the maternal blood levels taken at birth. The sampling period at 5 years of age shows a decline in *p,p’*-DDE to below maternal blood levels. However, comparisons of other contaminant levels in the 5-year-old sampling event show that levels of PCB153 are similar to those in maternal blood. Each follow-up year of biomonitoring shows a decline in levels of contaminants in the sample population (22, 23). Levels of PFOS in 7.5-year-old children were lower than levels in Cohort 1 children at 7 years of age. PFOA levels were only slightly lower in Cohort 3 than in Cohort 1 (25). Total Hg levels were much lower in the 5- and 7.5-year-old children than in cord blood at parturition. Cord blood levels of total Hg were also lower than levels seen in the previous two cohorts.

Mothers in Cohort 5 (Table X) were born 20 years later than the mothers of Cohort 1, and should have experienced pre- and postnatal exposure to PCBs only after the PCB phase-outs (26), whereas mothers in Cohort 1 would have accumulated body burden during the years that PCBs were in use. Even the 10 year difference in birth years between Cohort 3 and Cohort 5 mothers may affect the body burden accumulated. This may be indicated in the data with levels of all PCBs lower in Cohort 5 mothers than Cohort 3 mothers. And compared with Cohort 3, Cohort 5 maternal blood levels of *p,p’*-DDE were lower. Cohort 5 children at 1.5 years of age had blood concentrations very similar to maternal blood concentrations at birth, but with slightly higher *p,p’*-DDE. However, Cohort 5 children at 1.5 years of age had lower *p,p’*-DDE than 1.5 year-olds in Cohort 3. Cohort 5 children were also the first cohort to have PFOS and PFOA tested in blood at age 1.5 years. Blood levels of total Hg were the lowest of the four cohorts.

**Table X:** Time series of blood POPs concentrations from the Faroe Islands Cohort 5 (2007–2009)

	2007–2009	2009–2011
	Mothers	Children
Mean age (range)	30.7 (17.2–49.4)	1.5 (1.4–1.7)
Sample size	n=500	n=363
*p,p’*-DDE	131 (6.0–1,517)	180 (15–4,414)
PCB153	91.2 (1.0–694)	105 (15–1,214)
PFOS	–	6.5 (1.4–28.3)
PFOA	–	2.9 (0.5–22.5)
Total Hg	4.6 (0.8–44.5)^a^	1.4 (0.1–21.3)

All participants were Faroese women with their children born between 2007 and 2009. Data presented as geometric means (range). POPs in µg/kg plasma lipid. PFCs and metals in µg/L whole blood.

a Cord blood.

## Norway

The northern Norway mother-and-child contaminant cohort study (MISA study) was designed to investigate maternal concentrations of OCs and metals. The study was conducted between 2007 and 2009 and included pregnant and delivering women (n=516). A suite of OCs, five toxic metals and five essential elements were analysed (27).

Low maternal concentrations of contaminants were generally observed in the MISA study participants (Table XI). Data are presented only in cases where detection frequencies were greater than or equal to 70%. Some contaminants were not reportable due to the coefficient of variation exceeding 70% and concentrations being too low. Comparing the northern Norway MISA data from 2006 to 2008 with data previously reported (2, 18, 19), the recent geometric mean of *p,p’*-DDE is almost half that reported in 2004 for Bodø, a town in northern Norway, although the range is wider (28). For PCB153, while the geometric mean for MISA study participants is slightly higher than that reported in 2004 for Bodø, the geometric means for participants from both Bodø and MISA show levels almost half those observed in 1996 in Kirkenes (18, 28). Levels of Cd, Hg and Pb have declined since 1994–1995 (18) in pregnant women, which indicates that foetal exposure has also declined. However, Se levels are also lower than those in 1994–1995, which could indicate a reduction in the consumption of marine mammals and fish. Age reached significance only for Hg, while an inverse association was observed between parity and Hg. The observed concentrations for Pb and Cd suggest exposures from hunting traditional foods and smoking, respectively (29).

**Table XI:** Concentrations of PCBs and OCs in pregnant women (early pregnancy) from northern Norway (mean parity=0.9 (0–4))

	2006–2008
Mean age (range)	30.6 (18–43)
Sample size	n=515
*trans*-Nonachlor	2.8 (0.6–17.6)
*p,p’*-DDE	38.7 (10.9–351)
HCB	9.6 (3.5–53.3)
PCB153	24.8 (5.3–201)
Total Hg	1.2 (0.1–6.6)
Pb	7.4 (2.2–25.8)^a^
Cd	0.2 (0.04–2.7)
Se	84.7 (58.2–128)^b^

Data presented as geometric means (range), in µg/kg plasma lipid. Average values presented for compounds with detection frequencies ≥70%.

a n=280;

b n=281.

The MISA study showed links between traditional and store-bought food consumed and measured serum concentrations of PFCs. In MISA study participants, levels of PFOS were greater than any other PFCs, followed by PFOA (Table XII). Berg et al. (30) demonstrated that women consuming more marine food had significantly elevated concentrations of PFOS and other PFCs. Women who consumed a large amount of game had higher concentrations of PFNA, while elevated concentrations of PFOS were detected in high consumers of white meat. There was also a relationship between the consumption of beef and salty snacks and higher PFOA concentrations. However, the strongest significant predictor of all the investigated PFCs was parity, which resulted in lower maternal levels (30). Total months of breastfeeding were significantly associated with lower serum concentrations of PFOS and PFOA, across parity groups. Age was positively associated with PFNA and PFDA.

**Table XII:** Serum concentrations of PFCs (µg/L) in the northern Norway MISA study group (mean parity=0.9 (0–4))

Mean age (range)	31 (18–43)	
Sample size	n=391	
	Median	Mean (range)
ΣPFOS	8.0	8.8 (0.3–35.8)
PFOS linear	4.7	5.1 (<LOD–19.1)
PFOS branched	3.4	3.7 (<LOD–18.2)
PFOA	1.5	1.7 (0.3–11)
PFNA	0.6	0.7 (0.2–4.4)
PFDA	0.2	0.3 (0.05–2.3)

Data show arithmetic mean (range) for PFCs with detection frequencies greater than 50%.

## Sweden

Several biomonitoring studies have been undertaken in Sweden. A biomonitoring report commissioned by the Swedish Environmental Protection Agency (EPA) was a follow-up to the health related environmental monitoring (HAMI) that began in 1994 to analyse concentrations of Hg in women from Sweden's northern, southern and western regions (31). Additional small investigations of contaminant levels in the Swedish population were also done.

**Fig. 1:**
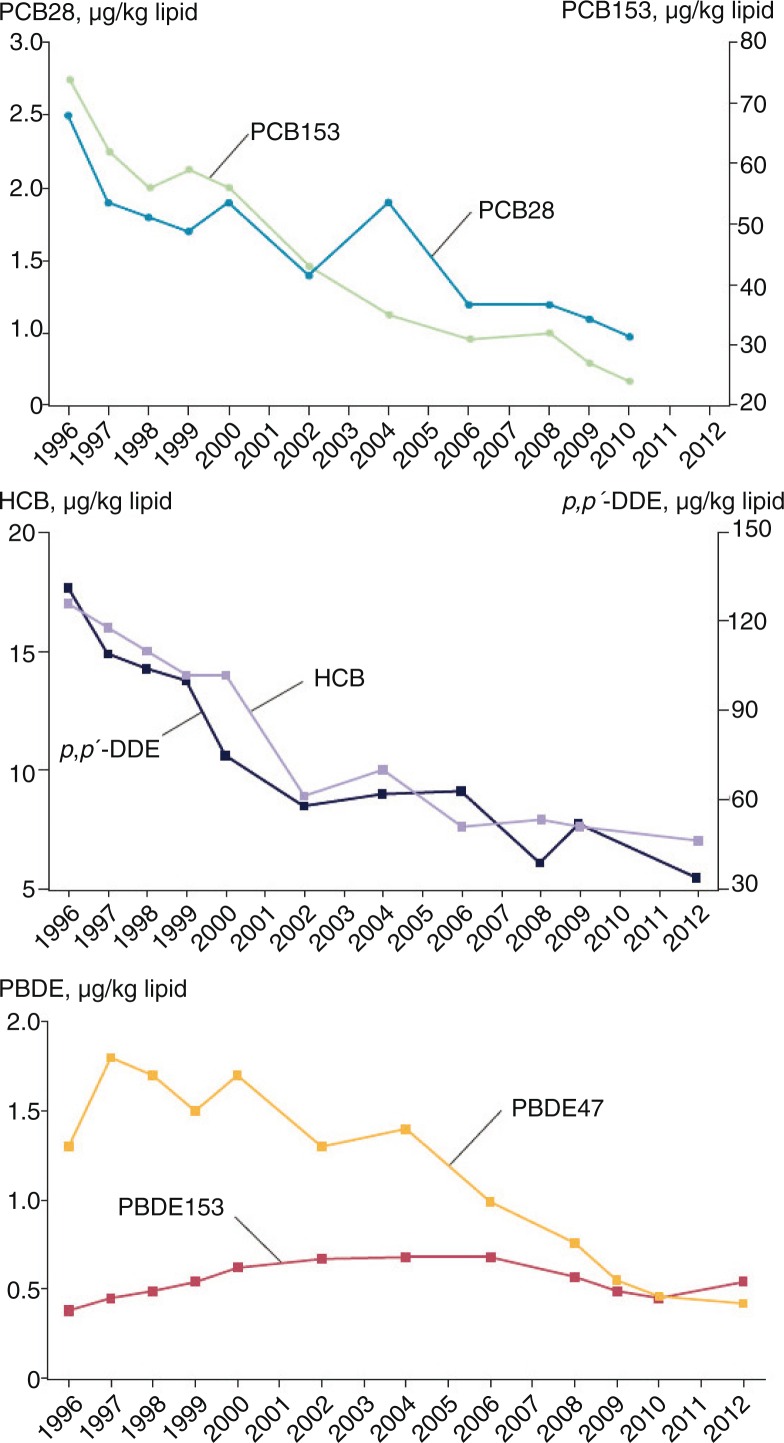
Trends in POPs concentrations in breast milk samples from Swedish first-time mothers (µg/kg lipid). Samples were collected 3 weeks after delivery. Data are presented as median concentrations (34).

**Fig. 2:**
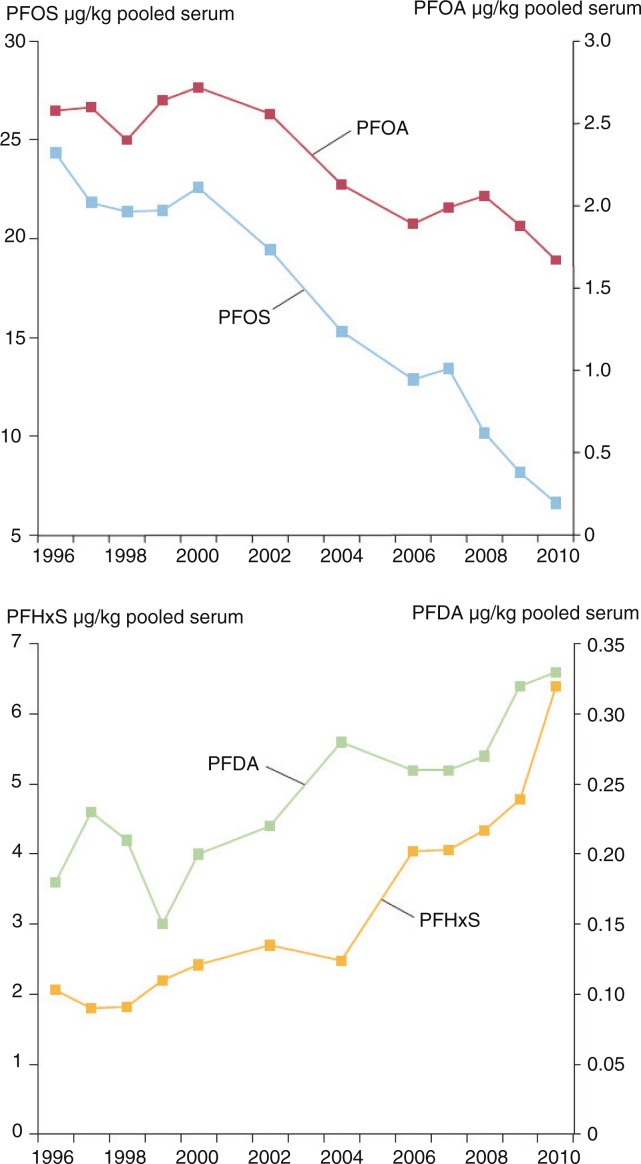
Trends in PFC concentrations (µg/kg pooled serum) in blood samples from nursing Swedish first-time mothers; blood samples were taken 3 weeks after delivery. Three pools per year were analysed, with serum from 5 to 25 individuals in each data pool. Data are presented as geometric means (33,34).

A time series from 1996 to 2012 of POPs (Fig. 1) in breast milk samples from Swedish first-time mothers in Uppsala shows a steady decline in all contaminants analysed except PBDE153 (32). PBDE153 fluctuated only marginally over the time series, reaching its highest concentration in 2004–2006, with an unclear pattern in recent years. A similar time series for PFCs (Fig. 2) was analysed in blood samples from Swedish first-time mothers, between 1996 and 2010 (33, 34). Both PFOS and PFOA have a general declining trend, with few data points that fall outside that trend; however, both PFHxS and PFDA show an increase over time (35–38).

In the follow-up HAMI study, a comparison was made of Hg in blood and hair from pregnant women in Sweden's north, south and west (Table XIII) (31). Mercury concentrations were higher in blood in western Sweden, with a median level of 1.2 µg/L whole blood, which is likely mainly due to the higher fish consumption in that area (31, 39). Despite women from southern Sweden eating more fish per week than those in Västerbotten, Hg levels in blood were comparable, and blood MeHg levels were higher in Västerbotten.

**Table XIII:** Median concentrations of Hg in blood (µg/L) in pregnant women in northern, western and southern Sweden

	Year	Sample size	Age (median)	Hg	MeHg	Fish (≥once/week) (%)
Västerbotten (North)	2003–2004	96	28	0.6	0.5	38
Gothenburg and Lysekil (West)	2001–2002	99	30	1.2	0.7	62
Hässleholm and Simrishamn (South)	2002–2003	100	30	0.6	0.3	50

## Finland

Breast milk has been sampled in Finland as a part of a follow-up study coordinated by the World Health Organization on levels of PCBs in human milk. The Finland component carried out a large population-based study, which recruited consecutive women giving birth in one of the maternity clinics from each of southern, central and northern Finland, the latter being the only Arctic population (40). New data for maternal blood concentrations of OCs and metals are not available.

The observable trend in southern Finland for PCB153 levels in breast milk indicates a decline from 1987 to 2010 (Table XIV). A decline is also seen in levels in central Finland, which also has lower levels of PCB153 than southern Finland on a year-by-year basis. Levels in northern Finland are only available for 2005 and 2010; however, these resemble those for central Finland for the same years. No change is evident from these two time points in the north. PBDE levels also show a declining trend from 2000 to 2010 for all three regions. Levels do not vary widely between regions.

**Table XIV:** Trends of contaminants in breast milk from first-time mothers in southern Finland (non-Arctic), central Finland (non-Arctic) and northern Finland (Arctic)

	1987	1993–1994	2000	2005	2010
Southern Finland					
Mean age (range)	26.9 (20–37)	27.9 (19–36)	29.2 (22–35)	28.7 (20–39)	31.2 (19–37)
Sample size	47	14	29	39	32
PCB153	113 (47.8–373)	85.2 (24.3–148)	38.7 (14.6–80.7)	24.8 (9.2–80)	22 (7.8–54.1)
PBDE^a^	–	–	2.9 (0.8–16)	2.3 (0.6–31.8)	1.3 (0.3–4.9)
Central Finland					
Mean age (range)	25.4 (19–34)	27 (18–39)	28.3 (19–36)	27.3 (19–40)	30.2 (19–36)
Sample size	37	28	31	40	19
PCB153	92.5 (38.9–209)	52.4 (18.2–110)	30.5 (10.5–72.7)	17.9 (4.8–65.1)	16.3 (7.2–50.3)
PBDE^a^	–	–	3.1 (0.6–13.5)	2.2 (0.5–11.3)	1.6 (0.5–6.6)
Northern Finland					
Mean age (range)				26.3 (21–41)	27.3 (20–32)
Sample size				11	20
PCB153	–	–	–	19 (9.3–50.9)	18.7 (6.8–58.1)
PBDE^a^	–	–	–	2.5 (1.4–6.3)	1.6 (0.6–3.8)

Data presented as geometric means (range) in µg/kg lipid. (40,41; Kiviranta, personal communication, 2014).

a PBDE47+PBDE99+PBDE100+PBDE153+PBDE209. PBDE, polybrominated diphenyl ethers.

## Russia

This section presents a follow-up study conducted in Russia involving the Chukotka 2001–2003 birth cohort reported by AMAP (2) and the 2007 follow-up involving biomonitoring sampling and investigation of health effects (42). As a part of the international KolArctic project (KO467), samples were taken in 2013 and 2014 in the Pechenga District of Murmansk Oblast in north-western Russia, near the Norwegian border. The sampled population included pregnant women, non-pregnant women and adult men (43).

Maternal blood Pb levels decreased by 21% between 2001–2003 and 2007 (Table XV), although the range broadened to include much lower and much higher levels in 2007. Maternal Hg levels remained essentially the same between the two sampling periods. This suggests a continuous exposure to Hg, probably through fish and seafood. Blood Pb levels in children have not changed, whereas concentrations of Hg in blood decreased by 31%. There may be differences in Hg exposure between mothers and their children; however, due to the relatively short half-life of MeHg in the body (44), it cannot be determined from these results whether the potential differences are the result of long-term behaviour or if there were more recent changes in exposure prior to the 2007 sampling.

**Table XV:** Maternal, cord and child blood concentrations of metals from coastal Chukotka, Russia (2001–2002 and 2007)

	Maternal (n=17)		Cord (n=17)	Child (n=17)
			
	2001–2002	2007	2001–2002	2007
Mean age (range)	24.6 (15–33)			5.5
Total Hg	1.6 (0.5–3.9)	1.6 (0.5–4.8)	1.4 (0.5–3.3)	0.9 (0.5–2.7)
Pb	37.5 (18.3–76.8)	29.6 (4.9–137)	37.6 (14.3–78.3)	38.2 (6.9–102)

Data presented as geometric means (range) in µg/L whole blood.

Levels of POPs in pregnant women were consistently lower than that of non-pregnant women and men (Table XVI) (43). Levels of POPs were also consistently lower in women than in men.

**Table XVI:** Concentrations of POPs in pregnant women, non-pregnant women and men in the Pechenga District of Murmansk Oblast, 2013–2014

	Pregnant women	Non-pregnant women	Men
Geometric mean age (range)	28.2 (16–41)	44.2 (26–65)	39.2 (27–54)
Sample size	50	17	33
*p,p’*-DDE	102 (16–1,221)	141 (39.4–538)	167 (51.6–940)
HCB	18.2 (5.3–252)	33.3 (12.8–85.1)	40.6 (10.9–189)
PCB153	12.2 (1.3–56.7)	27.5 (8.5–61)	47.9 (21.6–141)

Data from the KolArctic Project (KO467). Data lipid-adjusted (µg/kg plasma lipid) and presented as geometric means (range), including age.

## Discussion

**Fig. 3:**
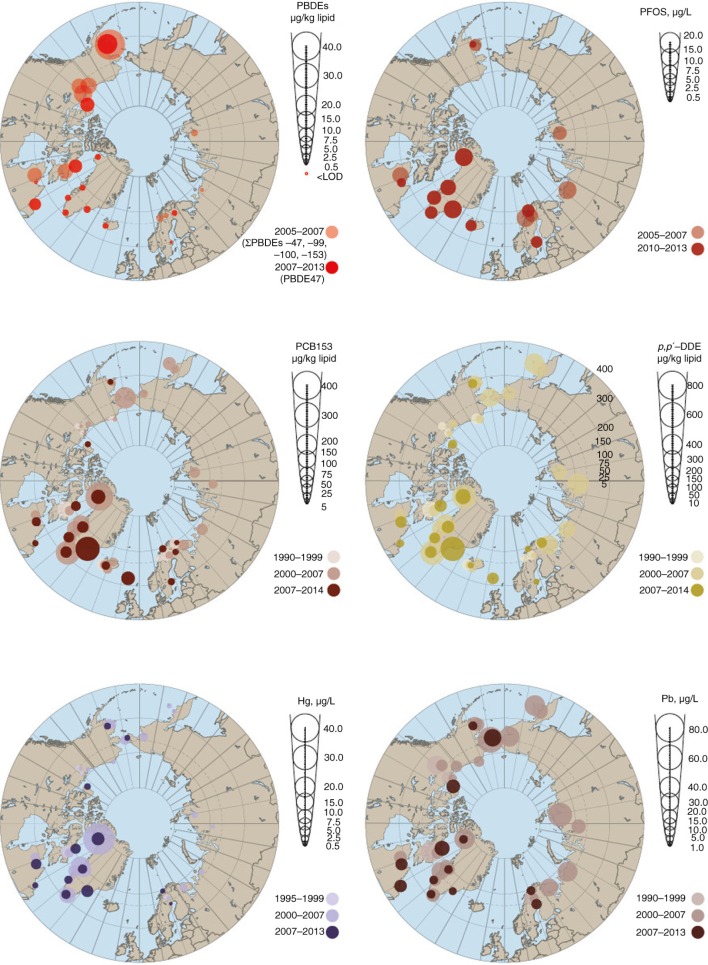
Circumpolar concentrations of PBDEs, PFOS, PCB153, *p,p’*-DDE, Hg and Pb. Unless otherwise indicated, POPs and PBDEs are presented in µg/kg plasma lipid, PFCs in µg/L and metals in µg/L whole blood. Data for Alaska, Faroe Islands, Sweden (except metals) and coastal Chukotka (Russia) are derived from maternal blood. Data for the Inuvialuit Settlement Region (NWT, Canada), Nunavut (Canada) and Nunatsiavut (NL, Canada) are derived from the blood of women of childbearing age. Data for Nunavik (QC, Canada), Greenland, Iceland, Norway, Sweden (metals only) and the Pechenga district (Russia) are derived from the blood of pregnant women. Data for Sweden (PBDE47, PCB153 and *p,p’*-DDE) and Finland are derived from breast milk.

Greenland remains one of the Arctic areas with populations still experiencing high levels of POPs. Eastern Greenland populations were found to have the highest levels of *trans*-nonachlor, *p,p’*-DDE, PCB153, HCB and PFOS (Fig. 3). Compared across the Arctic, Greenland populations had the highest measured levels for more POPs than any other Arctic country, with the exception of PBDEs. The maternal population in Alaska had the most elevated levels of PBDE47 and PBDE99, although the levels of PCB153, HCB and *trans*-nonachlor were among the lowest in the Arctic. Populations in Nunavik (Canada) had the second highest level of *p,p’*-DDE. However, Canada also has some of the lowest levels of PCB153, as shown for populations from the Inuvialuit Settlement Region and Nunatsiavut, and for PFOS and PFOA measured in populations from Nunavik. The Faroese PCB153 levels were elevated, sometimes by three- to fourfold, compared with populations in other countries, except eastern and northern Greenland. Populations sampled in Reykjavik (Iceland) had the highest PFOA levels; however, they also had the lowest levels of *p,p’*-DDE, and levels of PCB153, *trans*-nonachlor, PBDEs and PFOS were also among the lowest.

Sampled Swedes and Norwegians had the next highest levels of PFOA, while levels of PFOS in Swedish populations are among the lowest in Arctic countries with data. Norway also had the lowest levels of *trans*-nonachlor, and some of the lowest levels of *p,p’*-DDE, PCB153 and HCB. Levels of *p,p’*-DDE and PCB153 in the pregnant women of the Pechenga District of Murmansk (Russia) are among the lowest reported here.

The highest levels of total Hg were observed in the blood of women of childbearing age (WCBA) (including pregnant women) from Nunavik in Canada (Fig. 3). WCBA from northern and eastern Greenland had slightly lower levels of total Hg in blood, at 8.0 and 7.0 µg/L whole blood, respectively. The lowest levels occurred in women from western Greenland, Sweden and Norway, with levels between the limit of detection and 1.2 µg/L whole blood.

Russian maternal blood from Chukotka taken in 2007 had the highest circumpolar levels of Pb at 29.6 µg/L whole blood. Women from Nunavut, Inuvialuit Settlement Region and Nunatsiavut region in Canada all had levels ranging from 18 to 27 µg/L whole blood, which was only slightly higher than levels found in 2002–2004 in women from eastern and mid-western Greenland (4), although recent levels in Greenlandic women are much lower at 6.0 to 7.0 µg/L whole blood. Low levels were also found in Yu'pik mothers in Alaska (7.4 µg/L whole blood) and Norwegian women (7.44 µg/L whole blood).

Selenium levels were not measured in pregnant women or WCBA in all circumpolar countries. The highest levels were found in women from the Canadian Arctic. Nunavut and Nunavik women had geometric means of 286 and 280 µg/L whole blood, respectively. The lowest mean blood concentrations for Se were found in women from Norway and Alaska. Cadmium levels were also not measured in all circumpolar countries, although the highest levels were again found in women from the Canadian Arctic and Greenland. The lowest levels occurred in women from Norway and Alaska.

In Russia and the Faroe Islands, levels of contaminants in children aged 5–7 years matched with levels of their mother's blood at parturition. Using calculated partition ratios, levels of certain contaminants in cord blood are expected to range from approximately 29 to 74% of levels found in the mother's serum (45). It is expected that pregnancy and breastfeeding lead to a displacement of contaminants for mothers (40–42). However, this also translates into the possibility of ongoing exposure for children during early childhood, during critical developmental stages, which may lead to adverse effects (46). This is why the focus on managing contaminant exposure for WCBA and pregnant women remains important, until levels of contaminants decline in sources of exposure.

As indicated in the Canadian, Greenlandic and Russian data sets, there were many cases of lower levels of POPs and OCs in women, and WCBA, than in men. This may be an indicator of the success in reaching the target population with the risk communication messages (47), or indicate a dietary shift from traditional foods to market foods. The lower levels in younger women could also reflect the age difference between groups, where older men or women may have higher body burdens. However, it also highlights a potential issue that men may not be receiving similar dietary advisories or appropriate information to make dietary choices for their own health. Studies are beginning to examine the potential contribution of epigenetic changes in sperm due to exposure to contamination, including contaminants of concern under the Stockholm Convention (48, 49). These epigenetic changes could result in adverse effects in children due to damage to the sperm cells. However, even without the concern about transmissible damage to germ cells, it is known that high body burdens of these contaminants of concern can lead to health effects in all adults (46).

Comparing levels in this assessment with other biomonitoring activities that occur globally, it is clear that some Arctic populations are still experiencing elevated levels of exposure to certain contaminants of concern, compared to populations elsewhere (Table XVII). For example, blood Hg concentrations are elevated in all countries where data are available, except in Sweden, where levels are comparable to those reported in the Canadian Health Measures Survey (CHMS) and the NHANES from the USA. The CHMS is representative of the general Canadian population that does not include the Canadian Arctic (50, 51). NHANES is also a national survey, representing the non-Arctic general population of the United States (52). Lead is also elevated in certain Arctic regions, especially in the Chukotka Peninsula of Russia, and Nunavik and Nunavut in Canada, but other Arctic countries have levels similar to those in the non-Arctic biomonitoring results. Levels of DDE in eastern Greenland populations are higher than those found in other countries. However, levels of DDE in all females aged 12 and above from the USA are higher than in pregnant women from the ACCEPT project in the rest of Greenland, and women from other Arctic countries. WCBA sampled in Nunavik (Canada) and mothers in the Faroe Islands have elevated levels compared with the CHMS. Levels of DDE in pregnant women from Russia's Murmansk region are comparable to those found in Canadian women aged 20–39 years from the CHMS.

**Table XVII:** Concentrations of selected contaminants across Arctic countries, compared with biomonitoring in non-Arctic areas

	USA^a^	CAN^b^	GRL^a^^,^^c^	ISL^a^	FRO^a^	NOR^a^	SWE	FIN	RUS	CHMS	NHANES
DDE	82.7	130^a^	587	36	131	38.7	34^d^^,^^e^	–	102^a^	102^f^	241^g^
PCB 153	14.8	40^a^	288	34	91.2	24.8	24^d^^,^^e^	18.7^d^	12.2^a^	8.2^f^	19.7^g^
PBDE 47	19.8	<LOD^a^	2.4	1.7	–	–	0.4^d^^,^^e^	1.6^d^^,^^h^	–	10.8^f^	19.6^g^
PBDE 99	4.5	<LOD	1.3	<1.3	–	–	–	1.6^d^^,^^h^	–	N/C^i^	N/C^i^
PFOS	2.2	3.9^a^	15.8	6.2	–	8.0^e^	6.7^f^	–	–	4.4^f^	7.6^g^
Hg	2.2	5.2^a^	7.0	–	–	1.2	0.6^a^^,^^e^	–	1.6^f^	0.7^f^	0.7^g^
Pb	7.4	14^a^	6.0	–	–	7.4	10^e^^,^^f^	–	29.6^f^	8.5^f^	8.4^g^
Se	181	300^a^	140	–	–	84.7	–	–	–	190^f^	188^g^

Data presented as geometric means, in blood of pregnant women or WCBA, except for DDE and PCB153 in Sweden and Finland which are levels in breast milk. POPs presented in µg/kg plasma lipid and metals in µg/L whole blood. CHMS, Canadian Health Measures Survey; NHANES, US National Health and Nutrition Examination Survey.

a Pregnant women;

b Nunavik;

c Eastern Greenland;

d data are for breast milk from first-time mothers (g/kg lipid);

e median value;

f WCBA;

g data for all females, ages 12+ years;

h PBDE47 and PBDE99 results combined with other PBDEs;

i geometric mean not calculated as proportion of results below detection limit was too high to provide a valid result.

Comparisons of PBDEs and PFOS and PFOA concentrations in blood samples obtained in Arctic and non-Arctic countries may point to different exposure pathways. PBDE47 levels in the maternal population in Alaska exceed those tested under the CHMS or NHANES, and they far exceed those found in other Arctic countries. PBDE99 levels in all Arctic populations exceed those found in the general population tested by the CHMS and NHANES, in that a geometric mean could not be reliably calculated for this contaminant because so few of the analysed population in the CHMS and NHANES had levels above the detection limit. Populations in Greenland showed very high levels of PFOS, whereas concentrations found in pregnant women in Iceland and first-time mothers in Sweden are more comparable with the concentrations found in the CHMS and NHANES. Geographical differences in concentrations of PBDEs and PFCs between Arctic populations may also be due to large-scale differences in the movements of the air masses and ocean currents carrying pollutants into the Arctic via long-range transport, or animal migration (18, 53).

## Conclusion

Biomonitoring studies are an essential component of managing human health risks from exposure to environmental contaminants, including the ability to analyse the risks and benefits for human populations which consume traditional food. Biomonitoring activities are currently ongoing in the eight Arctic countries.

Maternal transfer of POPs and metals to infants is decreasing as maternal levels decrease (e.g. Faroe Islands), resulting in levels in newborns presently lower than what they were 20 years before. However, data show that children are still accumulating higher levels of contaminants in their early years, with levels decreasing later in adolescence and early adulthood, potentially due to dilution through growth, changes in dietary preference and/or other factors.

Levels of most POPs have declined since 1986, although certain PFCs do not show such decline. Changes in PBDE levels follow a pattern different from most other POPs, suggesting an alternative exposure route, such as dust from indoor furnishings (54). PBDEs may not accumulate in the same way as other POPs, such as PCBs in the food chain, or a combination of sources of exposure from food and consumer products or furnishings may cause a different accumulation pattern.

Blood Hg concentrations are still elevated in samples from Greenland, parts of Canada, and the Faroe Islands, although they are lower now in maternal blood compared to that found in 1986. Levels in Norway and Sweden have decreased and are now equivalent to those found in non-Arctic North America, such as for US women aged 16–49 years in 2009–2010 (0.86 µg/L) (55) and Canadian women aged 20–39 years from the general population in 2007–2009 (geometric weighted mean 0.70 µg/L) (56).

Blood Pb concentrations are still elevated in parts of Russia and Canada, whereas levels in other Arctic countries have declined. As suggested by Fillion et al. (57), there may be other important sources of exposure in addition to lead shot in country food, such as paint and ammunition dust contributing to the Pb load in house dust.

Precaution, including dietary advice (47), is still important for WCBA and pregnant women in the Arctic. Despite elevated levels, certain high concern contaminants (e.g. *p,p’*-dichlorodiphenyltrichloroethane (DDT), PCB153, HCB, Hg) in the presented Arctic populations are continuing to decrease relative to previous years. It is possible that this is evidence that international and national risk management of long-range transport contaminants may be having a positive effect, an indicator being the decline in measured body burdens after considering the population demographic and birth years for sampled populations. While local dietary advice and dietary transitions may also be resulting in decreased current exposure (47), for the more persistent POPs, older individuals born prior to the regulatory actions may nonetheless be expected to have a greater lifetime accumulation of the more persistent POPs. Differences in levels seen between men and women may also be evidence that specific dietary recommendations for WCBA and pregnant women may be reaching their target populations, although this may depend on the length of time of dietary advisory compliance required to see changes in body burden. Moreover, a woman's reproductive behaviours, such as breastfeeding and number of children born, can have additional impacts on her chemical body burden. Although the observation that decreasing levels of contaminants in women are resulting in a decline in contaminant exposure for the foetus, there have been fewer studies over time involving men; thus, it is less clear whether adult men are protected from the potential health effects of elevated body burdens of contaminants of concern.

Despite global action through the Stockholm Convention to reduce the production and use of POPs, contaminants are still being transported to and recycled within the Arctic environment (53, 58). In addition to long-range transport as a source of contaminants to the Arctic, there is evidence that global climate change may affect the cycling of contaminants within the Arctic environment (59), with the potential for release of contaminants currently held in soil, permafrost or ice, although few changes have been seen in the Arctic food web to date (58, 60). Nevertheless, changes in the structure and dynamics of the Arctic food web, especially in relation to species forming part of the traditional diet, could have implications for contaminant levels in Arctic populations, with associated impacts on human health. Although time series data sets indicate a current decline in concentrations of most POPs in Arctic biota, particularly PCBs and DDT, and contaminants such as PBDEs and PFOS that prior to 2000 appeared to be increasing now appear to show either no trends or a decrease (61), the potential implications for human health highlight the clear need to continue biomonitoring contaminants of concern. Further biomonitoring will also aid in measuring the success of international risk management strategies for reducing risk for all Arctic and vulnerable populations.
